# Automating bone loss measurement on periapical radiographs for predicting the periodontitis stage and grade

**DOI:** 10.3389/fdmed.2024.1479380

**Published:** 2024-10-10

**Authors:** Nazila Ameli, Monica Prasad Gibson, Ida Kornerup, Manuel Lagravere, Mark Gierl, Hollis Lai

**Affiliations:** ^1^ School of Dentistry, Faculty of Medicine and Dentistry, University of Alberta, Edmonton, AB, Canada; ^2^ Faculty of Dentistry, University of Indiana, Indianapolis, IN, United States; ^3^ Faculty of Education, University of Alberta, Edmonton, AB, Canada

**Keywords:** grade, periodontitis, stage, u-net, YOLO

## Abstract

**Background:**

The aim of this study was to develop and evaluate an automated approach for segmenting bone loss (BL) on periapical (PA) radiographs and predicting the stage and grade of periodontitis.

**Methods:**

One thousand PA radiographs obtained from 572 patients were utilized for training while a separate set of 1,582 images from 210 patients were used for testing. BL was segmented using a U-Net model, which was trained with augmented datasets to enhance generalizability. Apex detection was performed using YOLO-v9, focusing on identifying apexes of teeth to measure root length. Root length was calculated as the distance between the coordinates of detected apexes and center of cemento-enamel junction (CEJ), which was segmented utilizing a U-Net algorithm. BL percentage (ratio of BL to the root length) was used to predict the stage and grade of periodontitis. Evaluation metrics including accuracy, precision, recall, F1-score, Intersection over Union (IoU), mean absolute error (MAE), intraclass correlation coefficients (ICC), and root mean square error (RMSE) were used to evaluate the models’ performance.

**Results:**

The U-Net model achieved high accuracy in segmenting BL with 94.9%, 92.9%, and 95.62% on training, validation, and test datasets, respectively. The YOLO-v9 model exhibited a mean Average Precision (mAP) of 66.7% for apex detection, with a precision of 79.6% and recall of 62.4%. The BL percentage calculated from the segmented images and detected apexes demonstrated excellent agreement with clinical assessments, with ICC exceeding 0.94. Stage and grade prediction for periodontitis showed robust performance specifically for advanced stages (III/IV) and grades (C) with an F1-score of 0.945 and 0.83, respectively.

**Conclusion:**

The integration of U-Net and YOLO-v9 models for BL segmentation and apex detection on PA radiographs proved effective in enhancing the accuracy and reliability of periodontitis diagnosis and grading.

## 1 Introduction

Periodontitis is a multifactorial and microbiome-associated inflammatory disease that occurs in the dental supporting tissues [periodontium, which includes gingiva, periodontal ligament, cementum, and alveolar bone presenting as bone loss (BL)] (1–3). Progression of the disease can adversely affect oral and systemic health and result in tooth loss, reduction of masticatory performance (4) as well as having association with diabetes (5) and coronary artery disease (6). Thus, periodontitis and its complications will impose substantially negative effects on oral health-related quality of life (OHRQoL) (4, 7). Moreover, early detection and diagnosis of periodontitis can help in preventing the consequent costly and invasive dental treatment (3).

In clinical settings, the assessment of periodontal conditions involves visual and tactile examinations. The gold standard for examination includes measurements of periodontal pocket depth (PPD), bleeding on probing (BOP), and clinical attachment loss (CAL) (8). Radiographs are employed to confirm diagnosis and treatment plans. However, variations in probe tip diameter, angulation, probing force, and intra-examiner differences can result in divergent outcomes. Additionally, in instances of mild attachment loss or when determining the subgingival localization of the cementoenamel junction (CEJ), accurately determining CAL is challenging due to the difficulty in ascertaining CEJ location. In such cases, precise and reliable assessment relies on the interpretation of radiographic bone levels radiographically (8, 9). Alveolar BL, is defined as any distance from CEJ to the alveolar bone crest that is greater than 2 mm (10).

Despite the improvements in the quality of image and resolution over the past decade, interpreting dental imaging is primarily and subjectively conducted by the trained dentist based on the individual's judgement and experience (11–13). Inconsistencies in their interpretation may result in misdiagnosis and, in the process of periodontitis evaluation, may lead to wrong measurement of BL (14). Currently, deep learning (DL) techniques are being widely applied for quick evaluation of dental images (15, 16), without subjective interpretations.

Traditional methods of automated medical image analysis include large amounts of rule-based algorithms or manual preprocessing methods that are time-consuming with low quality and poor generalization capability (17–19). This underwent changes with the introduction of convolutional neural networks (CNN) algorithms using DL, which allow for direct interference, recognition and classification of medical images (20, 21). Due to its demonstrated efficiency in the field of image understanding (image segmentation, classification, localization and detection) through feature extraction of input data, it is a widely used technique for solving medical image understanding (22).

Image segmentation is the process of identifying key components of an image and separating the image into individual sections or regions- is a fundamental task in medical image processing (23). Several DL models have been introduced for medical image segmentation like the U-Net and mask-RCNN (24). Most image segmentation DL models are based on CNNs (25). Specifically, the U-Net architecture has been thoroughly studied for biomedical image segmentation due to its ability to produce highly accurate segmented images using limited training data (26, 27). The popularity of this algorithm is evident from its widespread adoption across major primary imaging techniques, including computed tomography scans, magnetic resonance imaging, x-rays, and microscopy (27).

An alternative method that employs CNN principles is You Only Look Once (YOLO), which is mainly designed to precisely identify objects in real time (28). A complex generalized approach toward object identification, YOLO combines concurrent processing of information at different convolutional layers with up-and-down sampling approaches similar to U-Net. One of the characteristics of YOLO is to extract features from the entire image and predict bounding boxes (28). In the literature, older versions of YOLO have been widely used in the field of dental radiology to detect mandibular fractures in panoramic radiographs (29), primary and permanent tooth detection on pediatric dental radiographs (30), detection of cyst and tumors of the jaw (31), periodontal BL detection (32), and detection of impacted mandibular third molar teeth (33). However, new versions of YOLO-v9, released in January 2024, demonstrate superior performance with respect to throughput and computational load requirements (34), and provide a network architecture that requires lower computing and training requirements, hence providing a more effective feature integration method, more accurate object detection performance, a more robust loss function, and an increased label assignment and model training efficiency (35).

While numerous studies have employed image mining techniques to measure BL and classify periodontitis, most have focused on BL at only two points: mesial and distal (24, 35–37). Additionally, these studies predominantly used panoramic radiographs (32, 38), despite evidence that periapical (PA) or bitewing radiographs provide more accurate BL evaluations (39, 40). This narrow focus on mesial and distal points risks underestimating the extent of BL, particularly in areas such as the furcation, where BL may be more pronounced.

Our study addresses these gaps by introducing an automated approach to segment BL across the entire width of each tooth, rather than limiting the analysis to mesial and distal points. By doing so, we ensure a more comprehensive assessment of BL, including in the furcation area, which is often overlooked. Furthermore, our study not only detects BL but also uses the segmented data to classify the stage and grade of periodontitis, a critical step that has been largely neglected in previous studies relying solely on image data. Additionally, we utilized the latest versions of U-Net and YOLO models, which offer superior performance in identifying key points for measurements and have not been extensively applied in the context of periodontitis. Importantly, we employed PA radiographs, the standard imaging modality for accurate BL detection, rather than panoramic images. By integrating these advanced techniques, our study provides a more precise and reliable framework for the detection, segmentation, and classification of periodontal disease, thereby contributing to improved diagnostic accuracy and treatment planning.

## 2 Materials and methods

### 2.1 Study population

After obtaining the University Human Research Ethics Board approval under the code Pro00107743, PA radiographs belonging to 210 patients collected from the Periodontal Graduate Program in the School of Dentistry between 2017 and 2021. A total of 1,582 images were included in the study for testing the model performance. Training was conducted for both BL segmentation and apex detection models utilizing a separate dataset comprising 1,000 PA images from 572 patients sourced from a private dental radiology office.

### 2.2 Bone loss segmentation

#### 2.2.1 Data preprocessing

**Figure 1:**
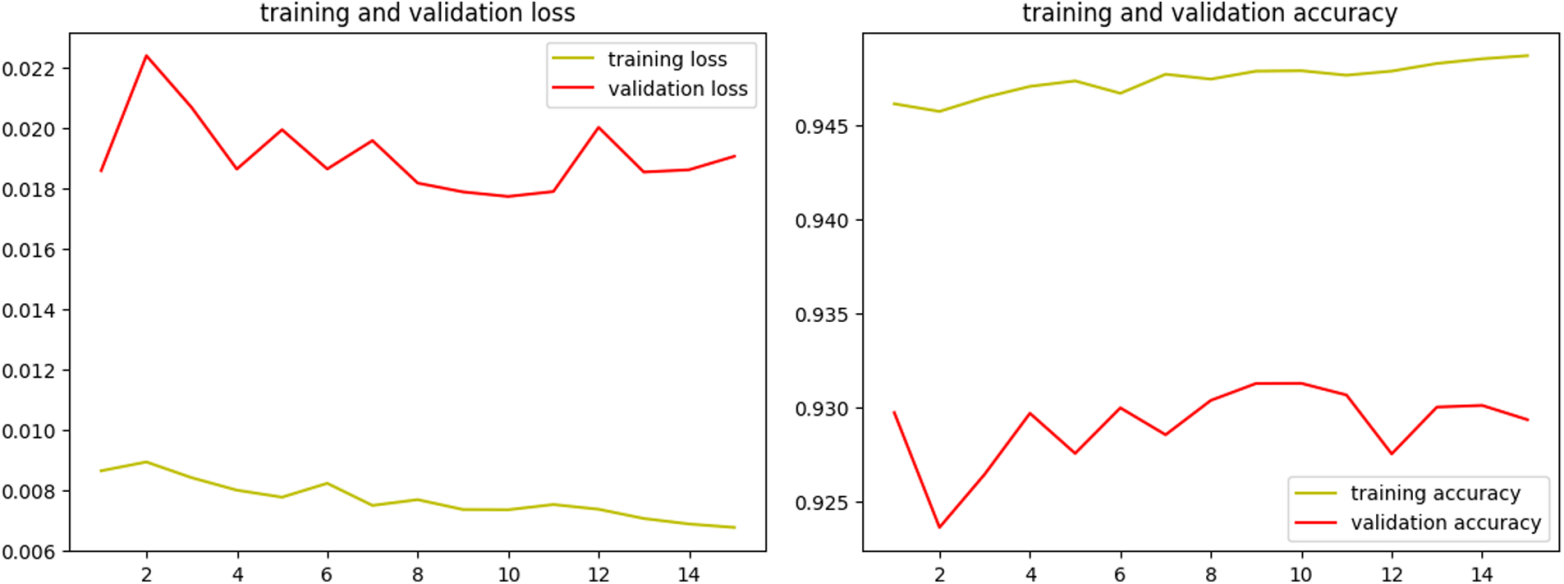
A polygon indicating the BL area (between CEJ and bone level).

To detect the amount of BL, the images were processed using an open-source tool called Roboflow (41) for annotation using polygons. Each radiograph was read by a dentist and a periodontist with more than 5 years of clinical experience. To manually determine the BL (the area between the CEJ of the teeth and bone level in tooth-bearing areas), a polygon passing through the CEJ of the teeth and the corresponding bone levels was drawn (Figure 1). The final label was determined based on consensus between the dentist and periodontist.

**Figure 2:**
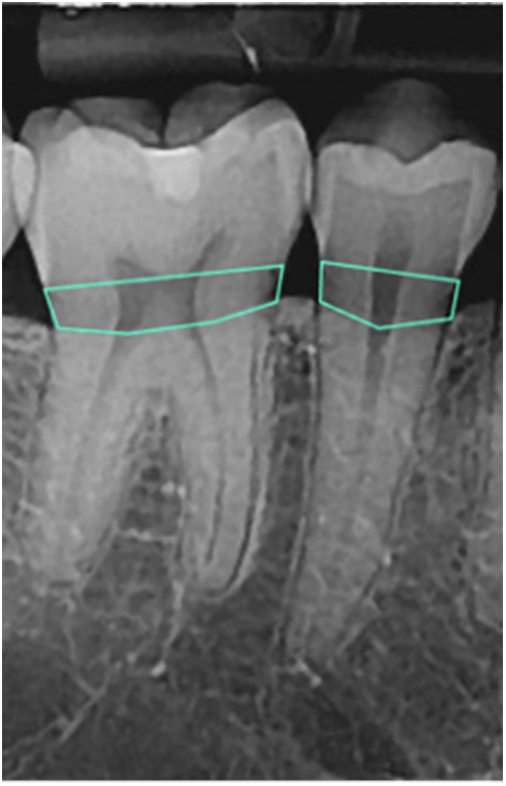
An example of a PA image with its corresponding mask.

The annotated area was then changed to a binary mask (BL as the mask area or region of interest and the background structures in black). The images and their corresponding binary masks were exported as Portable Network Graphics (PNG) format for further analysis (Figure 2). The image dataset was preprocessed for future analysis, which included resizing to 160*320 pixels and enhancement using histogram equalization technique.

#### 2.2.2 Data augmentation

Image augmentation continued after data splitting to ensure that data leakage would not occur. First, the total of 1,000 PA images was randomly split into three parts: training, validation, and testing in an 80:10:10 ratio (42, 43), respectively using scikit-learn library (44), then the augmentation was done through horizontal flipping and/or rotating at different angles (45) to increase out the dataset by two folds. The final result was a total of 1,600 PA images and their corresponding masks for training and 200 images and masks for validation.

#### 2.2.3 U-Net architecture for BL segmentation

Following data augmentation, the image dataset was normalized by dividing by 255 to scale down the pixel values of the images and their corresponding masks to a range between 0 and 1. The set of 1,582 images obtained from 210 patients referred to the university periodontal clinic were finally used to evaluate the model performance and its generalizability.

Different variations of U-Net (46) were trained and evaluated to find the best architecture and hyperparameter settings for the segmentation model. Finally, a U-Net model featuring 4 encoder blocks with increasing filters (64, 128, 256, 528), a bridge of a convolutional block with 1,024 filters, and 4 corresponding decoder blocks with decreasing filters (512, 256, 128, 64) was utilized for training. The model was trained with a batch size of 8 and a learning rate of 0.0001 over 15 epochs, using the Adam optimizer, ReLU activation function, and the Dice coefficient as the loss function.

#### 2.2.4 Evaluation of diagnostic outcome

Evaluation metrics include accuracy (the ratio of the correctly predicted observations to the total observations), recall (the ratio of true positives to the sum of true positives and false negatives), precision (the ratio of true positives to the sum of true positives and false positives), and F1-score (the harmonic mean of precision and recall (35). Also, the Intersection over Union (IoU) and area under receiver operating characteristics (ROC) curve were used to evaluate the model performance on the validation dataset. The magnitude of these metrics ranges from 0 to 1 indicating a perfect performance (47).

Additionally, the comparison was made between the maximum amount of BL predicted by the model for each tooth and the measurements obtained by the dentist and periodontist using several metrics that are the standard metrics in understanding the efficiency of the model in terms of error rate, including the mean absolute error (MAE) and root mean square error (RMSE). Also, the intraclass correlation coefficient (ICC) (48, 49), which is a desirable measure of reliability that reflects both degree of correlation and agreement between measurements was used to assess the consistency and agreement among three raters (dentist, periodontist, and model predictions) concerning numerical measurements. For the MAE and RMSE the best value equals 0 while the worst value can be infinity (50). For ICC, values less than 0.5 are indicative of poor reliability, values between 0.5 and 0.75 indicate moderate reliability, values between 0.75 and 0.9 indicate good reliability, and values greater than 0.90 indicate excellent reliability (51).

### 2.3 Apex detection

#### 2.3.1 Data preprocessing

**Figure 3:**
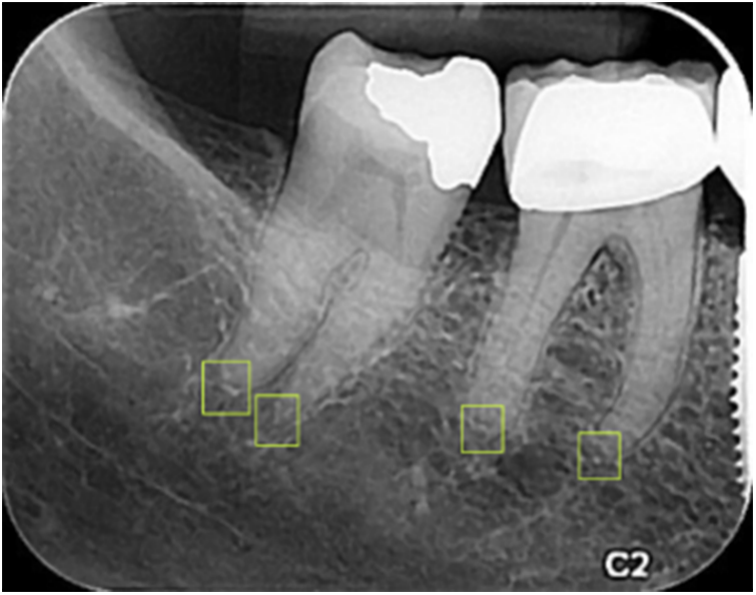
Annotating the apexes on PA images using bounding boxes.

A total of 1,000 PA images were imported into Roboflow (41) for annotation. Annotation was performed by delineating bounding boxes around the apices, ensuring that the center of each box corresponded to the apex's anatomical point. The annotations were carried out by a general dentist and subsequently reviewed and approved by a periodontist with over 10 years of experience (Figure 3).

Subsequently, the annotated images were exported in PNG format for subsequent analysis. The final determination of the bounding box was made through consensus between the dentist and the periodontist.

Furthermore, the image dataset underwent preprocessing steps in preparation for future analysis. This preprocessing included resizing all images to dimensions of 160 × 320 pixels. This standardized format ensures consistency and facilitates subsequent computational procedures, ensuring the compatibility of the dataset for further analysis and model training.

#### 2.3.2 Data augmentation

The data was split using Roboflow (41) splitting tool into 70% training, 20% validation and 10% test datasets. Image augmentation was done afterwards during model training to ensure that data leakage would not occur.

#### 2.3.3 YOLO-v9 architecture for apex detection

To detect the apexes of the teeth, the YOLOv9 model was utilized. Hyperparameters were defined to optimize training, with a learning rate set at 0.01. The model was trained for 75 epochs with a batch size of 16.

During training, various techniques were employed to enhance model performance and robustness. These included: data augmentation using techniques such as blur, median blur, grayscale conversion, and Contrast Limited Adaptive Histogram Equalization (CLAHE) to introduce diversity and variability in the training dataset. Additionally, optimization was performed using the Stochastic Gradient Descent (SGD) optimizer with a learning rate of 0.01. The training process was monitored using metrics such as box loss, class loss, and DFL (Detect From Language) loss, which were computed and evaluated across multiple epochs.

The training dataset consisted of annotated images and labels, which were scanned and preprocessed before training commenced. The dataset was divided into training and validation sets, with appropriate caching mechanisms implemented to optimize data loading and processing efficiency. Furthermore, performance evaluation was conducted periodically during training using metrics such as precision, recall, and mean Average Precision (mAP) calculated at various Intersection over Union (IoU) thresholds.

#### 2.3.4 Evaluation of diagnostic outcome

To evaluate the model performance and generalizability in the correct detection of root apexes, precision, recall, and mean average precision (mAP) were used on the validation dataset. Additionally, the model's performance on the new test dataset (1,582 images) was evaluated using MAE (the average magnitude of the errors between the predicted values and the actual values), RMSE (the square root of the mean of the squared differences between the predicted and actual values), and ICC (two-way random effect, single rater, absolute agreement) (48, 51) to compare the agreement in the coordinates of the apexes in the ground truth with those predicted by the model.

### 2.4 Measurement of maximum bone loss percentage

#### 2.4.1 Finding the maximum BL height

The maximum BL for each tooth on 1,582 new PA radiographs was independently measured by a general dentist and a periodontist, both with over five years of experience. These measurements were conducted using Adobe Photoshop 24.1.1, where the cursor was dragged to draw a line connecting the CEJ to the deepest bone level via the ruler tool. BLs less than 2 mm from the CEJ were not considered normal, as they were not detectable by the model.

To determine the maximum height of BL in every individual PA radiograph first, a custom Python function was developed to automate the process of quantifying BL height within the images. This function utilized established image processing techniques, including thresholding and connected component analysis, to identify regions of interest corresponding to areas of BL.

Upon thresholding the input image, connected component analysis was applied to identify distinct regions representing areas of BL (white areas in the masks). Each identified region was then enclosed within a bounding box, facilitating the extraction of its maximum height. This process ensures that the height measurement accurately reflects the extent of BL within the region of interest.

To evaluate the performance of the method in accurately quantifying maximum BL height across the dataset, the measured maximum BL heights by three raters were compared using the MAE, RMSE, and ICC utilizing the “pingouin” package in Python to assess the reliability or consistency of measurements taken by computer, dentist, and the periodontist.

### 2.5 Finding the root length

#### 2.5.1 Finding the coordinates of the segmented CEJ center

To accurately calculate the root length of lower and upper teeth from radiographs, a method was developed to locate the center point of the CEJ using OpenCV for image processing. Firstly, the mask images were preprocessed by thresholding to obtain binary images. The thresholding technique converts grayscale images into binary images, simplifying subsequent image analysis.

Subsequently, contours representing the segmented mask in the binary images were identified using the contour detection algorithm provided by OpenCV. Contours are continuous lines or curves that represent the boundaries of objects in an image. In this context, contours outlined the edges of the BL area in the mask images.

For each identified contour, the bounding rectangle enclosing the contour was calculated. From the bounding rectangle, the center point of the upper border of the segmented area for lower teeth, and lower border of the segmented area for the upper teeth were determined. This center point corresponds to the approximate location of the CEJ center, a critical anatomical landmark for measuring root length. The coordinates were then recorded for further analysis.

To evaluate the performance of the method in accurately locating the CEJ center points across the dataset, the values were compared to their ground truth using the MAE, RMSE, and ICC.

#### 2.5.2 Measuring the distance between CEJ and detected apex

To calculate the root length for finding the percentage of BL, the Euclidean distance formula - enables the calculation of the straight-line distance between two points on the image grid solely based on their pixel coordinates- was utilized. The formula encapsulates these computations:

Distance =  $\sqrt{{\left(X2-\;X1\right)}^{2}\;+\;{\left(Y2-Y1\right)}^{2}}$ in which, (x1, y1) and (x2, y2) denote the pixel coordinates of the two points respectively. (x1) and (x2) represent the x-coordinates and (y1) and (y2) represent the y-coordinates of the first and second points. By applying this formula, the exact distance between the obtained CEJ center point and the root apex were determined. Finally, using the maximum BL height and the root length for each individual tooth, the BL percentage was calculated.

Also, the BL percentage for each tooth on PA radiographs was independently measured by the same dentist and periodontist. These measurements were performed using Adobe Photoshop 24.1.1, where the cursor was used to draw a line connecting the CEJ to the root apexes (root length) with the ruler tool. The BL percentage was calculated by dividing the maximum BL by the measured root length. The BL percentages obtained from all three raters were then compared to evaluate consistency using RMSE, MAE, and ICC metrics to find the consistency in the BL percentage measurements.

### 2.6 Determining the stage and grade of periodontitis

The calculated percentage of the maximum BL was then used to categorize the patients’ periodontitis stage as stage I (<15%), stage II (15%–33%), and stage III/IV (>33%). Also, to assign the grade of the disease, the same value was divided by the patient's age. The outcome values less than 0.25 were assigned to grade A, between 0.25 and 1 were assigned to grade B, and values greater than 1 were assigned to grade C. To evaluate the final results, evaluation metrics (accuracy, precision, recall, and F1-score) were used to compare the output of this chapter with the ground truth stages and grades.

## 3 Results

### 3.1 Bone loss segmentation

#### 3.1.1 U-Net performance in segmenting Bl

The U-Net model achieved an accuracy of 94.9%, 92.9%, and 95.62% on training, validation, and test datasets, respectively for segmentation of BL.

**Figure 4:**
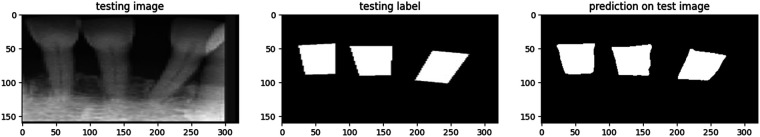
Training results of the U-Net model for segmentation of BL on PA radiographs.

Over the course of 15 epochs, the model demonstrated a consistent improvement in both training and validation performance, as indicated by the metrics of loss and accuracy. The learning rate remained constant at 1.0000e-04 throughout the training, ensuring a stable and gradual optimization process without drastic changes that could destabilize the learning dynamics. The results of the training process are shown in Figure 4.

In addition to the training and validation metrics recorded over the 15 epochs, the model's performance was further evaluated using several other metrics to provide a comprehensive understanding of its effectiveness. The Receiver Operating Characteristic (ROC) curve and Area Under the Curve (AUC) were used to assess the model's diagnostic ability for both validation and new test images.

**Figure 5:**
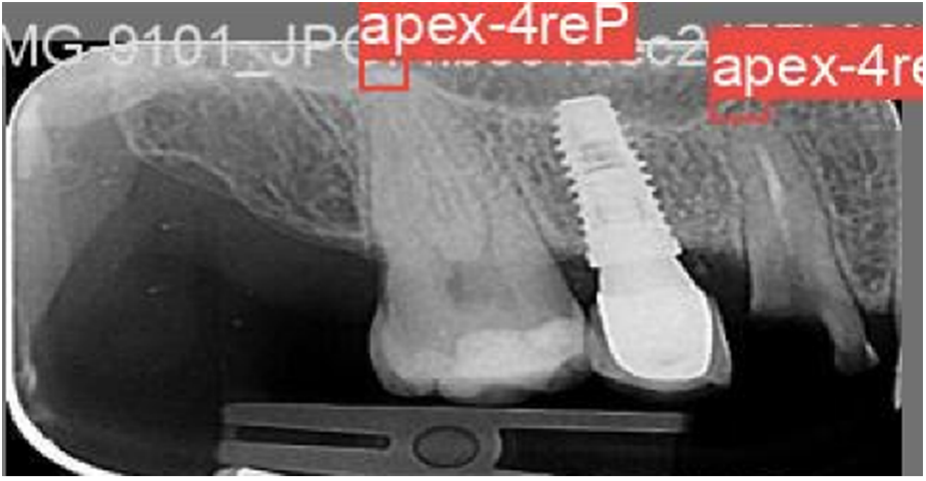
ROC curve and AUC, evaluating the model performance on the validation **(A)** and test **(B)** datasets.

The ROC curve plots (Figures 5A,B) the true positive rate (sensitivity) against the false positive rate (1-specificity) at various threshold settings, offering a graphical representation of the model's performance. The AUC provides an aggregate measure, with an AUC of 1 indicating a perfect model and an AUC of 0.5 indicating no discriminatory power (52). For the validation dataset, a high AUC value of 0.95 demonstrated the model's strong ability to segment the BL area correctly. Similarly, evaluating the ROC curve and AUC (0.90) on the test dataset confirmed the model's generalization capabilities. Consistent high AUC values for both datasets indicated that the model performed well without overfitting to the training data.

The Intersection over Union (IoU), also known as the Jaccard Index, was used alongside the DC to further evaluate the segmentation performance. IoU measures the overlap between the predicted and true segmentation masks by dividing the intersection of these masks by their union. IoU values also range from 0 to 1, with 1 indicating perfect segmentation where the predicted mask exactly matches the ground truth. This metric is particularly valuable for assessing how well the model predicts the boundaries and shapes of objects within an image. High IoU values confirmed the model's ability to accurately capture the details and contours of the segmented objects.

**Figure 6:**
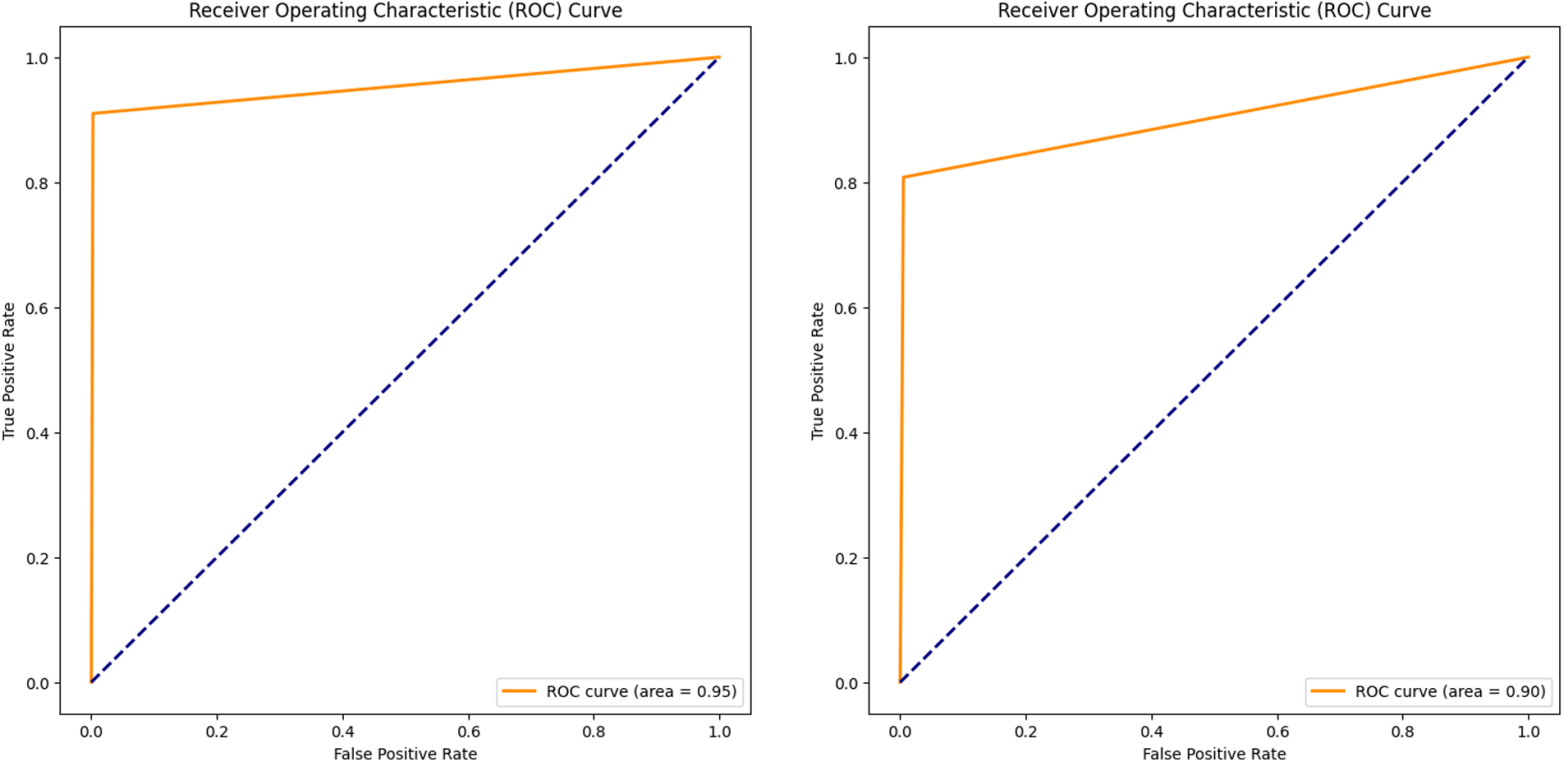
Model segmentation performance on the test dataset compared to the ground truth label.

The proposed model showcased a great performance across various metrics on the test dataset. Figure 6 represents a sample of the prediction made by the model for segmenting the BL area in a PA image. The overall accuracy of the model on the test dataset was 95.62%, indicating a high level of correctness in its segmentation task.

Table 1 depicts the evaluation metrics used for assessment of model performance on a set of 1,582 new PA images. According to Table 1, precision, measuring the proportion of correctly predicted positive pixels among all predicted positive pixels, was notably high at 97.26%. This suggests that the model exhibits a strong capability to accurately segment the regions of interest while minimizing false positives (53). However, the recall metric was comparatively lower at 63.90%. This indicates that while the model accurately identifies many positive pixels, it misses slightly more than 1/3 of the actual positive pixels present in the images. In segmentation tasks, recall reflects the model's ability to capture the entirety of the target objects, highlighting areas where the model may need improvement.

**Table 1:** Performance metrics of the proposed U-Net model on 1,582 new set of PA images for segmenting BL.

Metric	Value
Accuracy	95.51
Precision	97.26
Recall	63.90
F1-score	77.12
Intersection over Union (IoU)	81.69

The F1-score was recorded at 77.12%. This metric provides a balanced measure of the model's segmentation performance, considering both false positives and false negatives. The F1-score underscores the need for a trade-off between precision and recall, indicating areas where the model may excel in delineating object boundaries while still needing enhancement in capturing entire objects. The IoU was measured at 81.69%, indicative of the effectiveness of the model in capturing the relevant regions of interest within the images.

#### 3.1.2 CEJ centre localization

In assessing the consistency of locating the CEJ center of the teeth for measuring the BL percentage, a thorough analysis was conducted using the same set of 1,582 new test images. The CEJ center coordinates obtained through the application of OpenCV library on the segmented masks were compared against ground truth annotations, which were verified by a consensus between a dentist and a periodontist.

To quantitatively evaluate the agreement between the predicted and ground truth coordinates, RMSE and MAE metrics were used for both the X and Y coordinates (Table 2). Moreover, the ICC was utilized to assess the inter-rater reliability between the model predictions and the ground truth annotations. The ICC values for CEJ_X and CEJ_Y coordinates were calculated as 0.9468 and 0.9629, respectively, indicating excellent agreement between the model predictions and the ground truth (52).

**Table 2:** Assessment of the agreement between the predicted and ground truth coordinates of CEJ center points on the test PA images.

Metric	CEJ-X coordinates	CEJ-Y coordinates
RMSE	12.64	10.93
MAE	4.55	4.25

#### 3.1.3 Maximum bone loss measurement

The results revealed that the dentist and periodontist exhibited the highest RMSE (0.0700) and MAE (0.0570), indicating the greatest discrepancies between these raters. In contrast, the periodontist and model measurements showed the lowest RMSE (0.0617) and a moderate MAE (0.0507), reflecting the best agreement. Measurements between the dentist and the model demonstrated intermediate values, with an RMSE of 0.0637 and the lowest MAE (0.0446), indicating good but slightly lesser agreement than that between the periodontist and the model.

Also, ICC analysis was done to have a better understanding of agreement among 3 raters. The results indicated that there is a very high level of consistency among the three raters for the maximum BL measurement. Both individual and average raters’ scores showed strong agreement, with ICC values consistently above 0.94. This suggests that the ratings are reliable and that the maximum BL measurement done by model can be confidently used for further analysis or decision-making processes.

Pairwise ICC comparison was done to find the agreement between every two raters (Table 3). According to the table, the best agreement was observed between the periodontist and the model measurements with the highest ICC1 (0.945) and F-value (35.71), and the 95% confidence interval (CI) of [0.92, 0.96].

**Table 3:** Inter-examiner reliability in maximum BL measurements among periodontist, GP, and the model.

Raters	ICC	*F*-value	95% CI	*p*-value
Dentist vs. model	0.944	34.73	0.92–0.96	<0.001
Dentist vs. periodontist	0.937	30.88	0.91- 0.96	<0.001
Periodontist vs. model	0.945	35.71	0.92- 0.96	<0.001

### 3.2 Apex detection

**Figure 7:**
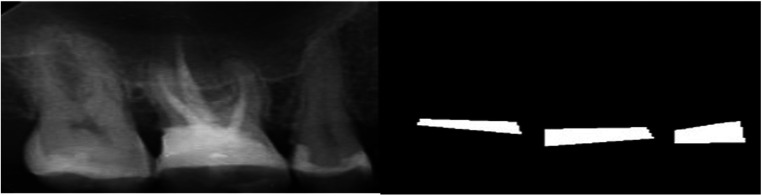
Training results of the YOLO-v9 model for apex detection.

**Figure 8:**
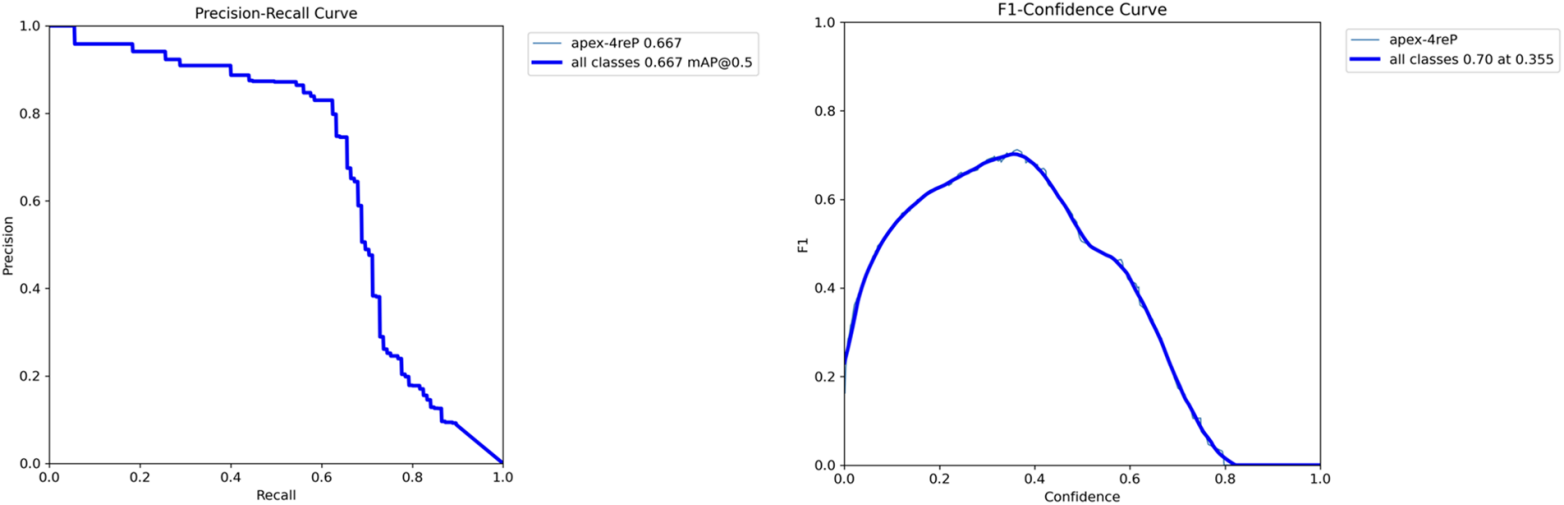
An example of the model performance in detecting apexes on PA images.

The YOLOv9 model, employed for detecting the apexes of teeth, was trained for 75 epochs, leveraging a range of hyperparameters to optimize its performance. The training results are shown in Figure 7. A sample of the model performance in detection of root apexes has been shown in Figure 8.

**Figure 9:**
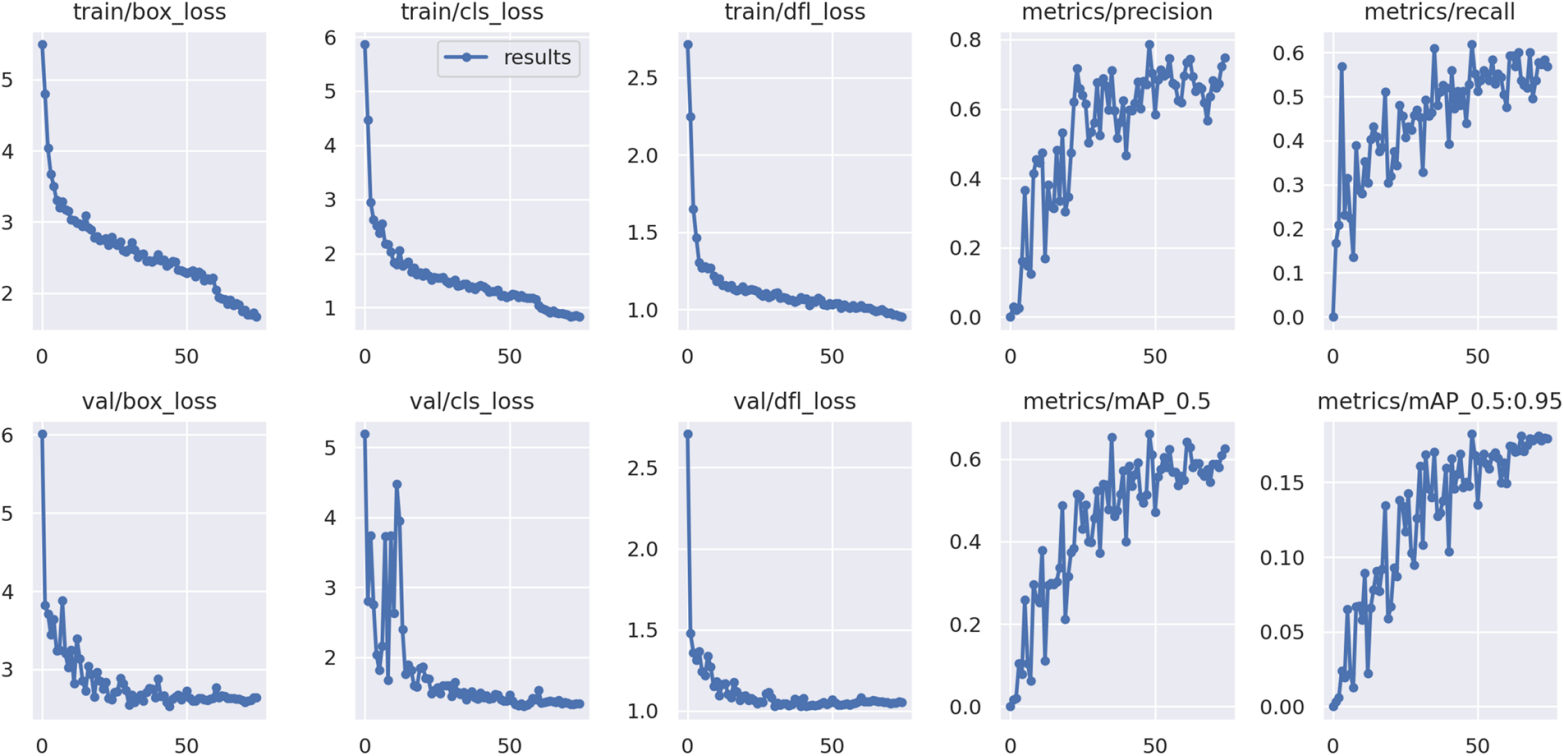
Performance analysis of YOLO-v9 model for apex detection: (**A**) precision-recall and F1-score curves.

The evaluation revealed promising results, with the model achieving a mAP of 66.7%. Additionally, the precision metric stood at 79.6%, indicating the model's accuracy in correctly identifying apexes. The recall metric, which measures the model's ability to capture all relevant instances, was recorded at 62.4%, showcasing less than two third of the true positives being correctly identified. Figure 9 demonstrates the precision-recall and F1-curves indicating the performance of the model in detecting root apexes.

To evaluate the performance of the YOLO-v9 model in accurately detecting tooth apexes, a comprehensive analysis was conducted using a new set of 1,582 images. The coordinates of the detected apexes by the model were compared against ground truth annotations, which were meticulously curated by a qualified dentist and subsequently confirmed by a periodontist to ensure accuracy and reliability.

To quantitatively assess the agreement between the predicted and ground truth coordinates, RMSE and MAE for both the X and Y coordinates were used (Table 4). Furthermore, the ICC measure for absolute consistency, was utilized to measure the inter-rater reliability between the model predictions and the ground truth. For the Apex_X coordinate, the ICC value was calculated as 0.9832 with a 95% confidence interval of [0.98, 0.99], indicating nearly perfect agreement between the model predictions and the ground truth. Similarly, for the Apex_Y coordinate, the ICC value was found to be 0.9936 with a 95% confidence interval of [0.99, 1.00], demonstrating excellent agreement between the model predictions and the ground truth.

**Table 4:** Assessment of the agreement between the predicted and ground truth coordinates of detected apexes on the test PA images.

Metric	CEJ-X coordinates	CEJ-Y coordinates
RMSE	1.25	0.03
MAE	0.15	0.01

### 3.3 Bone loss percentage

The results indicated that the model has higher consistency with the periodontist (lower RMSE values) compared to the dentist. Specifically, the RMSE (3.98) values for the periodontist and measurements made by the model comparison were lower than those for the dentist and model comparison (4.64), suggesting that the model's predictions align more closely with those of the periodontist. Additionally, the comparison between the periodontist and the dentist revealed that there is a moderate level of consistency between their measurements (RMSE = 4.28). However, the slightly higher RMSE values in the dentist and periodontist comparison indicated some variability in their BL percentage evaluations.

Additionally, the ICC analysis indicated an excellent consistency among the three raters for the BL percentage measurement. Both individual and average raters’ scores demonstrated excellent agreement, with ICC values consistently exceeding 0.94.

Pairwise ICC comparison was done to find the agreement between every two raters (Table 5). According to the table, the pair with the best agreement was the model vs. periodontist, with the highest ICC (0.9588) and F-value (47.5692), and a CI of [0.94, 0.97].

**Table 5:** Inter-examiner reliability in BL percentage measurements among periodontist, GP, and the model.

Raters	ICC	*F*-value	95% CI	*p*-value
Dentist vs. model	0.943	34.33	0.92–0.96	<0.001
Dentist vs. periodontist	0.954	42.54	0.93–0.97	<0.001
Periodontist vs. model	0.959	47.57	0.94–0.97	<0.001

### 3.4 Prediction of periodontitis stage

The performance of the proposed method for predicting the stage of periodontitis was evaluated on a new test dataset comprising 210 samples (181 stage III/IV, 27 stage II, and 3 stage I). The overall metrics for stage prediction demonstrated promising results, as shown in Table 6. The method achieved a precision of 0.7805, an accuracy of 0.7952, a recall of 0.7952, and an F1-score of 0.7878. These metrics indicate a robust performance in identifying the correct stage of periodontitis across the test dataset.

**Table 6:** Metrics for individual stage and grade categories.

Stage/grade	Precision	Accuracy	Recall	F1-Score
I	0.0	0.0	0.0	0.0
II	1.0	0.19	0.19	0.32
III/IV	1.0	0.89	0.89	0.94
A	0.0	0.0	0.0	0.0
B	1.0	0.71	0.71	0.77
C	1.0	0.62	0.62	0.83

The detailed analysis of the individual stage categories revealed varying degrees of prediction accuracy, as summarized in Table 6. For stage III/IV, the method exhibited excellent performance with a precision, recall, and accuracy of 1.0, 0.8950, and 0.8950, respectively, resulting in a high F1-score of 0.9446. This indicates that the model was particularly effective in identifying advanced stages of periodontitis.

Conversely, the method's performance for stage II was significantly lower, with precision, recall, and accuracy of 1.0, 0.1923, and 0.1923, respectively, and an F1-score of 0.3226. This suggests difficulties in correctly identifying stage II cases, potentially due to overlapping features with other stages.

For stage I, the method failed to correctly identify any cases, resulting in precision, recall, accuracy, and F1-score all being 0.0. This highlights a significant limitation in the model's ability to predict early-stage periodontitis, likely due to the insufficient number of training samples for stage I, with only three samples available. To enhance the model's performance in this category, further refinement and the inclusion of a larger, more representative dataset for early-stage periodontitis are necessary.

### 3.5 Prediction of periodontitis grade

The performance of the proposed method for predicting the grade of periodontitis was assessed on a separate new test dataset of 209 samples (8 with grade A, 58 grade B, and 144 grade C). The overall metrics for grade prediction showed a precision of 0.67, an accuracy of 0.66, a recall of 0.66, and an F1-score of 0.66. These results indicate a reasonable ability to predict the grade of periodontitis, although there is room for improvement.

An in-depth examination of the individual grade categories provided further insights, as detailed in Table 6. For grade B, the method performed excellently with precision, recall, and accuracy of 1.0, 0.7083, and 0.7083, respectively, and an F1-score of 0.8293. This reflects a strong capability in identifying grade B periodontitis cases.

For grade C, the method also showed good performance with a precision, recall, and accuracy of 1.0, 0.6207, and 0.6207, respectively, and an F1-score of 0.7660. This suggests that the model is fairly accurate in predicting more advanced grades of periodontitis. However, the method struggled significantly with Grade A predictions. The precision, recall, accuracy, and F1-score for grade A were all 0.0, indicating a complete inability to correctly identify any cases in this category. This underscores the need for further increase in the size of data by utilizing a larger, more diverse training dataset to capture the nuances of early-grade periodontitis.

## 4 Discussion

This study aimed to enhance the accuracy and efficiency of diagnosing and grading periodontal disease using advanced models. Specifically, a U-Net model for segmenting BL on PA images and a YOLO-v9 model for detecting the apexes of teeth were employed. These methods were chosen for their recent advancements and high performance in medical imaging tasks (26, 27, 34). Comparing our results with those from existing literature highlights both our achievements and the areas where our approach offers distinct advantages.

The proposed U-Net model for BL segmentation achieved high performance in segmenting BL, with an accuracy of 95.62%, precision of 94.67%, recall of 66.06%, F1-score of 78.16%, and IoU of 80.74%. These metrics are competitive with those reported in similar studies, such as the work by Shon et al. (54), where the U-Net model was used to segment the boundary of periodontal BL (PBL) and CEJ on panoramic radiographs. In their approach, the U-Net algorithm was applied separately to detect PBL and CEJ boundaries by creating segmentation masks for each structure. The model was trained independently for PBL and CEJ segmentation, with training conducted for 30 epochs and a batch size of 16 on a dataset of 1,044 panoramic radiographs. Shon et al. reported training and validation accuracies of 98.6% and 98.9% for PBL and CEJ segmentation, respectively.

In the present study, PA radiographs, which are the standard for detecting periodontal disease (55–57), were utilized. The method for segmenting the boundaries of the BL and CEJ involved a single U-Net model, applied to segment the boundaries of both structures. This approach simplifies the segmentation process and is tailored to the specific radiographic modality used in periodontal diagnosis.

Chang et al. (58) also applied a hybrid DL approach for detecting the radiographic bone level on panoramic radiographs. The aim of their study was to develop an automated method for diagnosing PBL (of individual teeth) for staging the periodontitis according to the 2017 World Workshop criteria (2) on dental panoramic radiographs using the DL hybrid method. They applied a modified CNN from the Mask R-CNN based on a feature pyramid network (FPN) and a ResNet101 backbone (59) to detect the PBL and CEJ on 330 panoramic radiographs which were increased by 64 times using the augmentation techniques. Similar to the approach by Shon et al. (54), the model was applied twice to segment the boundaries of PBL and CEJ separately. Their results demonstrated a Jaccard index of 0.92, pixel accuracy of 0.93, and DC of 0.88 for PBL detection. For CEJ level segmentation, the Jaccard index, pixel accuracy, and DC values were 0.87, 0.91, and 0.84, respectively. These results are comparable to those obtained in our study, highlighting the effectiveness of automated models for detecting PBL on radiographs. However, in the present study, PA radiographs were used instead, and a single U-Net model was trained to segment both the PBL and CEJ. This approach represents a more efficient method for determining the extent of PBL on dental radiographs.

Ezhov et al. (60), applied U-Net with CNN architecture to segment the alveolar BL on 1,135 CBCT scans. The PBL sensitivity and specificity were 0.95 and 0.97, respectively with the highest values obtained for severe PBL (0.93 AND 0.99, respectively). Although CBCT can provide three-dimensional information, there are still some limitations caused by artifacts, noise and poor soft tissue contrast (61). Additionally, CBCT is not routinely prescribed for the detection of periodontitis due to its relatively high radiation dose compared to PA radiographs.

In a recent study conducted by Li et al. (62), the authors systematically reviewed papers to identify the application of DL for the classification of periodontitis and assess the accuracy of this approach. In terms of dental imaging modalities, the studies included primarily utilized PA images, panoramic images, and CBCT images for periodontitis classification. PA radiograph images capture the teeth and surrounding alveolar bone, thus providing comprehensive information on RBL. However, this modality has a limited view, typically showing only three to four teeth per image (63). Regarding the task of classification using DL models, classical models such as U-Net and YOLO were often utilized in the included studies (46, 64), regardless of the specific diagnosis task chosen. The authors reported that U-Net has been proven to quickly and accurately identify targets in medical images and generate high-quality segmentation results. Additionally, the structure of U-Net can be flexibly adjusted according to the specific needs of the task (62, 65).

Several studies have utilized image mining approaches to detect the amount of BL for classifying periodontitis. While accurate identification and delineation of BL is crucial for periodontal treatment planning and monitoring disease progression, most studies have focused on measuring the BL at two points (mesial and distal) (14, 35–37). The novelty of this research lies in segmenting the BL across each individual tooth rather than focusing solely on mesial and distal points. This approach mitigates the potential for underestimating the extent of BL, providing a more comprehensive and accurate assessment specifically when the furcation is involved in posterior teeth.

In the context of object detection, the introduction of the YOLO models has further revolutionized the field, with applications across diverse contexts demonstrating remarkable performance relative to their two-stage counterparts (66). For detecting the apexes of teeth, the proposed YOLO-v9 model exhibited minimal error. The RMSE for the Apex-X coordinate was 1.25 and for the Apex-Y coordinate was 0.03, while the MAE was 0.15 and 0.01, respectively. This precision is critical for accurate root length measurements. The YOLO-v9 algorithm represents a significant advancement in the field of object detection, providing real-time performance with remarkable accuracy (34, 66).

The recent YOLO-v9 builds upon its predecessors by incorporating enhancements that further improve both accuracy and speed. Notable improvements in YOLOv9 include the integration of advanced backbone networks, which facilitate richer feature representation and enhanced context perception. Additionally, the introduction of PANet (Path Aggregation Network) enables effective feature fusion across different scales, leading to improved localization accuracy. Comparative analysis with previous YOLO versions and other object detection algorithms demonstrates the efficacy of YOLOv9 in achieving a favorable balance between accuracy and speed (34, 67, 68).

A systematic review conducted by Li et al. (62), has shown that various versions of YOLO, from YOLOv3 to YOLOv5, have been utilized for detecting the PBL and classifying the periodontitis stage on various dental images. Consistently, Uzun Saylan et al. (32), utilized a YOLO-v5 algorithm to detect the BL across 685 panoramic radiographs. In addition to general evaluation, models were grouped according to subregions (incisors, canines, premolars, and molars) to provide a targeted assessment. Their findings revealed that the lowest sensitivity and F1 score values were associated with total alveolar BL (0.75 and 0.76, respectively), while the highest values were observed in the maxillary incisor region (1 and 0.95, respectively). They concluded that YOLO architecture has significant potential in analytical studies detecting PBL conditions.

After obtaining information about the key points needed for calculating the BL percentage (coordinates of apex, CEJ center, and maximum BL) through the proposed U-Net and YOLO-v9 algorithms, the BL percentage was calculated by dividing the maximum BL by the length of the root, yielding high agreement with clinician assessments. The ICC values were 0.943 for dentist vs. model, 0.954 for dentist vs. periodontist, and 0.959 for periodontist vs. model, indicating strong reliability of the proposed model's measurements.

Additionally, the strong ICC values for the coordinates of detected apexes and segmented CEJs, indicating a high level of agreement between both models’ predictions and the ground truth values. This demonstrates the accuracy and reliability of the proposed integrated model in detecting these key anatomical landmarks, further validating its potential utility in clinical applications.

The stage and grade prediction results showed varying performance across different categories. For stage III/IV, the model achieved a precision of 1.0 and an F1-score of 0.945, indicating high accuracy for severe cases. However, performance for stage I was poor, with an F1-score of 0.0. This discrepancy is similar to challenges reported in other studies, such as the work by Chang et al. (58), who also found lower accuracy in classifying early stages of periodontitis. However, the results of the present study emphasize the need for more robust models and larger, more diverse datasets to improve early-stage detection. In grade prediction, the proposed model performed well for grades B and C, with F1-scores of 0.829 and 0.766, respectively. However, it failed to accurately predict grade A. In this study, the distribution of the test dataset was significantly imbalanced, with the highest frequency of teeth classified as stage III and grade B. While the imbalance in the training dataset was partially mitigated by augmenting the images of minority stages and grades, this issue needs further attention through additional data collection in future research.

The overall metrics for grade prediction were found to be lower compared to those for stage prediction. This finding aligns with the results of Ertas et al. (35), who reported lower clinical accuracy values for classifying the grade of periodontitis using panoramic images (64.5% vs. 88.2%). This could be explained by the fact that the progression of periodontal disease (grade) is influenced by multiple factors. While some individuals may develop severe periodontitis rapidly, others may maintain a mild stage of the disease throughout their lives. Additionally, the progression of periodontitis is less predictable in certain patients, necessitating diverse treatment plans. Established risk factors that accelerate BL include smoking and poorly controlled diabetes, alongside obesity, genetics, physical activity, and nutrition (69, 70).

In contrast to the staging of periodontitis, there are a few studies that determine the grade of periodontal disease solely based on BL percentage. Shon et al. (54), selected a YOLO-v5 architecture for detecting the teeth objects and classifying them according to the teeth numbering system. The detected tooth numbers were then integrated with the boundaries identified using U-Net to determine the length of the roots and stage of periodontal disease for each tooth. They reported that the integrated framework had an accuracy of 0.929, with a recall and precision of 0.807 and 0.724, respectively, in average across all four stages.

Similarly, Jiang et al. (36), applied a YOLO-v4 model integrated with U-Net to determine the stages of periodontitis according to the percentage of PBL. After segmenting each tooth using the U-Net model, YOLO-v4 was utilized to detect the 6 key points for each posterior tooth (CEJ mesial and distal, bone level mesial and distal, and root apexes mesial and distal), based on which the PBL% was mathematically calculated on a set of 2,560 panoramic radiographs. They concluded that the performance of the model was entirely acceptable, with an overall accuracy of 0.77, although it varied across different teeth.

Comparing our findings with existing literature, several differences and advantages of our approach become evident. In this study, the latest iterations of U-Net and YOLO models (YOLO-v9), were utilized, which have demonstrated superior performance in recent benchmarks. This choice likely contributed to higher accuracy and precision observed in the results, particularly in segmentation and apex detection tasks. The high ICC values and minimal errors in apex detection highlight the precision and reliability of the proposed models, making them suitable for clinical application. These metrics are consistent with or superior to those reported in studies using older model versions or alternative approaches.

Additionally, by integrating U-Net for segmentation and YOLO-v9 for detection, the authors leveraged the strengths of both models, resulting in a comprehensive approach to diagnosing and grading periodontal disease. This hybrid approach is supported by studies that have shown improved performance using combined models (58, 71). A novel hybrid framework combining DL architecture demonstrated high accuracy and excellent reliability in the automatic diagnosis of PBL percentage for individual teeth.

This study's methodology, which aligns well with clinical practices and provides high agreement with clinician assessments, underscores the practical applicability of our models. By using the most recent versions of U-Net and YOLO, this study benefits from the latest advancements in DL, resulting in higher accuracy and precision. In addition to evaluating segmentation and detection performance, we validated our models against clinician assessments, thereby providing a comprehensive evaluation of their clinical utility. This method utilized the percentage rate of BL to automatically stage and grade periodontitis, following the new criteria proposed at the 2017 World Workshop (2).

Oh et al. (72) conducted a study to identify discrepancies in periodontitis classification among dental practitioners with different educational backgrounds. The study included two cohorts: dental practitioners with periodontal backgrounds (n1 = 31) and those with other educational backgrounds (n2 = 33). The survey instrument featured three periodontitis cases (one with stage III grade C, one with stage II grade B, and one with stage IV grade B), along with guidelines for classification and a questionnaire comprising both closed and open-ended questions. The study evaluated the accuracy of correct classification and the agreement between the two cohorts. The findings revealed a fair level of agreement in periodontitis classification among practitioners from different educational backgrounds. The periodontal cohort demonstrated better accuracy in classifying the stage (71.33%) and grade (64%) compared to the non-periodontal cohort (61.67% and 49.33%, respectively). In comparison, the results of the approach in the present study show higher accuracy in determining the stage and grade of periodontitis than those observed in Oh et al.'s study involving human evaluators.

The findings of this study have significant implications for the field of dental diagnostics. The application of AI to automate the classification of periodontitis stages and grades can greatly enhance diagnostic accuracy and efficiency, ultimately leading to improved patient outcomes. By minimizing dependence on subjective interpretations and increasing the consistency of diagnostic assessments, these AI models hold the potential to revolutionize periodontal disease management. Moreover, integrating these techniques into Clinical Decision Support Systems (CDSS) can further augment clinical workflows and decision-making processes, providing real-time, evidence-based recommendations and enhancing overall patient care.

Recently, software tools like the electronic periodontal diagnosis tool (EPDT) and web-based PocketPerio application (73, 74) have shown to increase diagnostic accuracy in clinical settings. These tools enhance traditional methods by providing a systematic approach to data collection and interpretation. However, while these applications enrich clinical diagnosis, they may not match the scalability and efficiency of AI-assisted methods in handling large volumes of data and detecting subtle patterns indicative of BL. AI models, such as those employed in this study, offer the potential to not only match but also surpass these advantages by incorporating advanced image processing and ML techniques to refine diagnoses further.

### 4.1 Limitations

Radiographs, while invaluable in diagnosing periodontitis, have certain limitations. They primarily offer a two-dimensional representation, which can lead to underestimation of BL, particularly in the buccal and lingual areas. Additionally, radiographs cannot visualize soft tissue, which is crucial for assessing the entire periodontal condition. The inability to capture early inflammatory changes and the difficulty in distinguishing between old and active BL further limit their diagnostic capabilities. Despite these drawbacks, radiographs remain a cornerstone in periodontal diagnosis, especially when complemented by clinical examination and patient history.

However, advances in AI, like the ones proposed in this study, are particularly promising as they allow for the precise classification of periodontal disease stages and grades by analyzing radiographic images. Furthermore, AI can assist in standardizing interpretations, reducing observer variability, and processing large datasets rapidly, which can improve clinical decision-making.

Also, the quality of the images and annotations was crucial. High-quality images with precise annotations are necessary for training accurate models, and any inconsistencies or errors in annotations could lead to incorrect predictions and misclassification of periodontal disease stages and grades. To mitigate this, multiple annotators, including dental professionals with expertise in periodontal disease, were engaged to ensure accurate image annotations. Preprocessing methods such as Contrast Limited Adaptive Histogram Equalization (CLAHE) were used to standardize image quality and address variations.

It's important to note that while the proposed method demonstrated precise classification of the stage and grade of periodontal disease, comparable to clinical experts, it relies solely on image data. This method does not replace clinical judgment, as it does not consider important patient information such as medical and dental history. Integrating these aspects remains essential for a comprehensive diagnosis.

Despite the challenges posed by data imbalance, the results of this research are clinically useful. The high accuracies observed, especially in advanced stages of periodontitis, suggest that these AI models can provide reliable diagnostic support, even when faced with unbalanced datasets. Clinically, this means that AI can assist in identifying severe cases that require immediate attention, thereby improving patient management and outcomes.

Future research should focus on expanding the dataset to include a more diverse patient population and different clinical settings. This would help validate the generalizability of the models and refine their performance further. It is also important to compare various models including the transformer architectures and CNNs to provide insights into which approaches are most effective for predicting periodontal disease stages and grades based on PA radiographs.

## 5 Conclusion

This study demonstrates the significant potential of utilizing advanced ML models, specifically U-Net and YOLO-v9, for the accurate segmentation and detection of periodontal disease markers on PA images. The results indicate that these models can achieve high precision and reliability in identifying BL and detecting apexes, crucial for calculating BL percentages and subsequent disease staging and grading.

The high concordance between the proposed model's measurements and clinician assessments underscores the practical utility of these AI tools in real-world clinical settings. By leveraging the strengths of U-Net and YOLO-v9, the authors have developed a comprehensive approach that enhances the accuracy and efficiency of periodontal disease diagnosis and grading. This method can systematically and precisely assist dental professionals in diagnosing and monitoring periodontitis using PA radiographs. Consequently, it has the potential to significantly enhance dental professionals’ performance in the diagnosis and treatment of periodontitis. Future research should aim to expand the dataset size and diversity and refine the models further to improve early-stage detection capabilities.

## Data Availability

The data analyzed in this study is subject to the following licenses/restrictions: the data that support the findings of this study are available from the University of Alberta but restrictions apply to the availability of these data, which were used under license for the current study, and so are not publicly available. Data are however available from the authors upon reasonable request and with permission of University of Alberta. Requests to access these datasets should be directed to Nazila@ualberta.ca.
